# Supplementary material for: Assessment of cognitive screening tests as predictors of driving cessation: A prospective cohort study of a median 4-year follow-up
Source: PLoS One. 2021 Aug 20;16(8):e0256527. doi: 10.1371/journal.pone.0256527 (PMC8378690; doi:10.1371/journal.pone.0256527)
Supplement: Attachment — Submitted filename: Reviewers comment.docx [file pone.0256527.s001.docx]

Thank you for offering an opportunity to review an article titled “Assessment of cognitive screening tests as predictors of driving cessation: a prospective cohort study of a median 4-year follow-up”. This article investigated the driving cessation predictability based on various clinical tests in drivers aged 70 or higher. Overall, the manuscript is well written. However, there are still some concerns as stated below. Therefore, I would like to recommend a major revision for the current manuscript.

**Abstract**

Well written.

**Introduction**

1. Background information of clinical tests were well described.

2. Lack of discussion on driving cessation based on previous literature. For example, readers may also want to know when driving cessation usually happens, what the relationship between driving cessation and individual health looks like, what would the impact of driving cessation looks like from an individual perspective as well as societal perspective, etc. If authors can include an in-depth discussion (perhaps, create one new paragraph), it would enhance the current manuscript and help readers catch the importance of the manuscript more easily.

3. Study objectives are clearly presented.

**Methods**

1. All the clinical tests utilized in the study were clearly demonstrated.

2. Other methodologies are well described.

**Results**

1. It seems like driving performance (Table 1) was assessed by driving evaluation officers (?). Until Table 1 in the Results, I was not aware of how driving performance was assessed. If there are any relevant information how it was determined, the authors may want to add a short description in the methods.

2. Aside from the number of motor vehicle accidents, sometimes the number of citations (warnings, or traffic tickets) could contain valuable information of fitness to drive. If there was any, try to add them in the results. If not, the authors may want to add this point in the limitation section.

3. In the Table 1, participant classification depending on the three outcome categories of UFOV outcomes (low, moderate, high) are not presented. Was there any reason? Otherwise, this information will help readers understand the demographics.

4. The authors divided the whole group into two groups, healthy and unhealthy. What made the authors classify those two groups? While I was reading the paragraph (Lines 238 to 248). I was expecting to see any analysis (i.e., a survival analysis depending on healthy vs unhealthy status), but I do not think there is any in the current manuscript.

**Discussion**

Good summary of findings and good discussion of strengths, limitations, interpretation, and practical implications.

**Conclusion**

Well-written

**Minor comments**

Line 52: need a space between 1.16 and -.

Line 192: What is the class III?

Line 472: MoCA
